# Oncogenic codon 13 *NRAS* mutation in a primary mesenchymal brain neoplasm and nevus of a child with neurocutaneous melanosis

**DOI:** 10.1186/s40478-014-0140-8

**Published:** 2014-10-21

**Authors:** Francis Shih, Stephen Yip, Patrick J McDonald, Albert E Chudley, Marc R Del Bigio

**Affiliations:** Diagnostic Services Manitoba, Winnipeg, MB Canada; Department of Pathology & Laboratory Medicine, University of British Columbia, Vancouver, BC Canada; Section of Neurosurgery, University of Manitoba, Winnipeg, MB Canada; Department of Biochemistry & Medical Genetics, University of Manitoba, Winnipeg, MB Canada; Department of Pathology, University of Manitoba and Diagnostic Services Manitoba, Room 401 Brodie Centre, R3E 3P5 Winnipeg, MB Canada

**Keywords:** Melanosis, Brain tumor, Genetic mutation, Somatic mosaicism

## Abstract

A 28-month female with a clinical diagnosis of neurocutaneous melanosis and numerous intracranial abnormalities (including a right choroid plexus tumor and left hemimegalencephaly) presented with a rapidly expanding tumor in the left occipital cerebrum. Microscopic examination of the resected specimen revealed a myxoid mesenchymal neoplasm consisting of fusiform cells that were immunoreactive for vimentin, CD34, and P53 but no melanocyte markers. Focused amplicon deep sequencing on DNA extracted from the brain tumor and a cutaneous nevus revealed a heterozygous (c.37G > C; p.G13R) substitution in the *NRAS* gene. DNA sequencing of “normal” skin and buccal swab showed the identical *NRAS* change albeit at lower allelic frequency. Her parents did not harbor the *NRAS* mutation. The skin lesion, but not the brain tumor, had a *BRAF* mutation (c.1397G > T; p.G466V). A germline single nucleotide polymorphism in *MET* was found in the child and her father (c.3209C > T; p.T1010I). The findings suggest *NRAS* mosaicism that occurred sometime after conception and imply an oncogenic role of the activating *NRAS* mutation in both the brain and skin lesions in this child.

## Background

Neurocutaneous melanosis (NCMS; Mendelian Inheritance in Man MIM# 249400) is a rare congenital phakomatosis consisting of numerous giant cutaneous nevi along with extensive leptomeningeal melanosis. Approximately 100 cases have been described in the literature [1]. The pathogenesis of NCMS likely involves a morphogenetic error in which melanocyte precursors derived from the neural crest migrate abnormally and proliferate locally [2,3]. Most cases with an identifiable cause have a somatic gain-of-function mutation in codon 61 of the *NRAS* gene (MIM# 164790) located on chromosome 1p13 [4-7]. Approximately 30% of affected children have melanin deposits detectable in the leptomeninges or brain [8] and half have epilepsy [9].

We present a case of a female infant with NCMS who developed an unusual myxoid mesenchymal brain tumor. DNA sequencing showed shared mutations in codon 13 of *NRAS* in the melanocytic nevi and the brain tumor.

## Case report

### Clinical details

**Figure 1:**
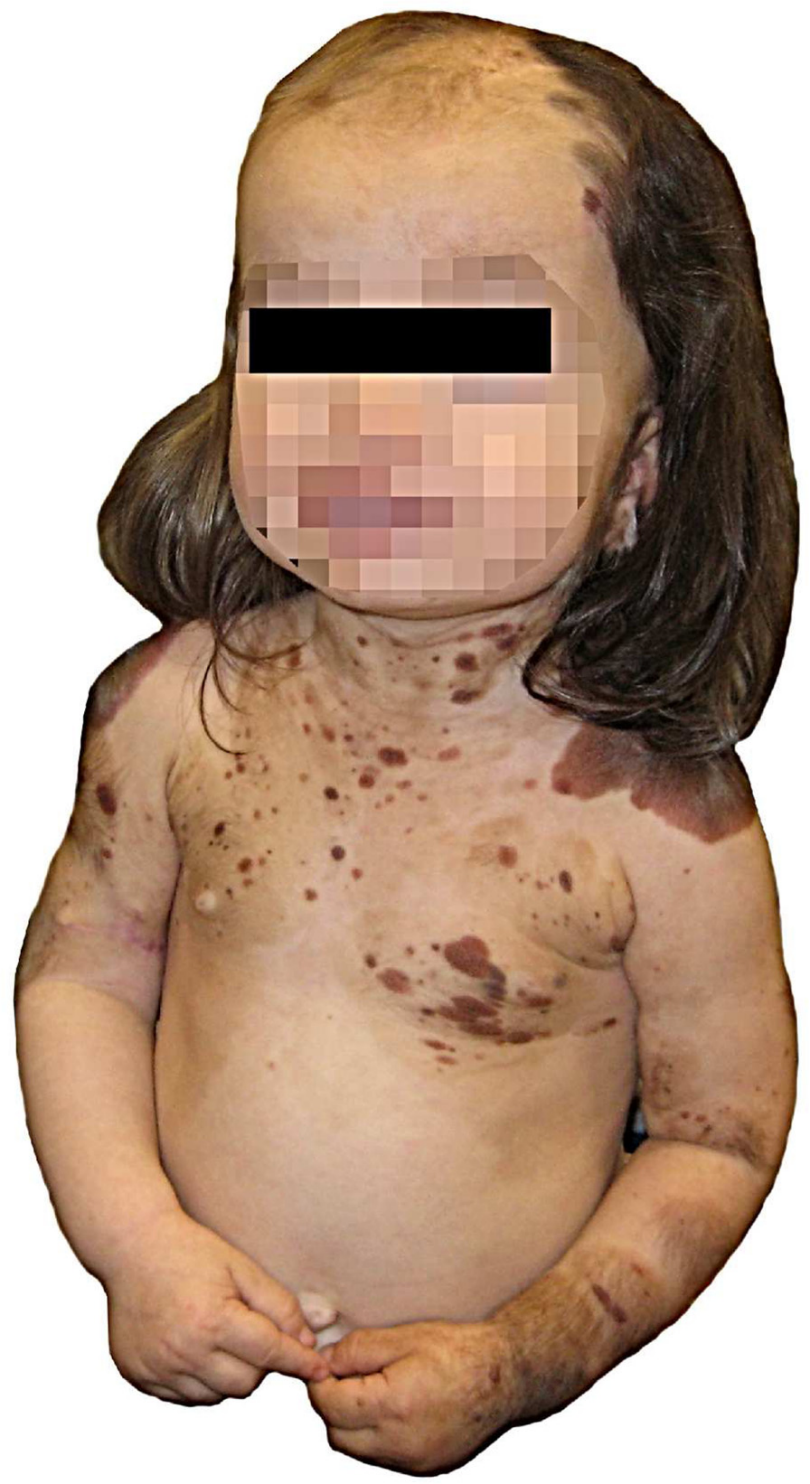
**Photograph of the patient at 14 months age showing giant congenital melanocytic nevi on each shoulder and numerous smaller satellite nevi on the neck, scalp, arms, and upper trunk.**

**Figure 2:**
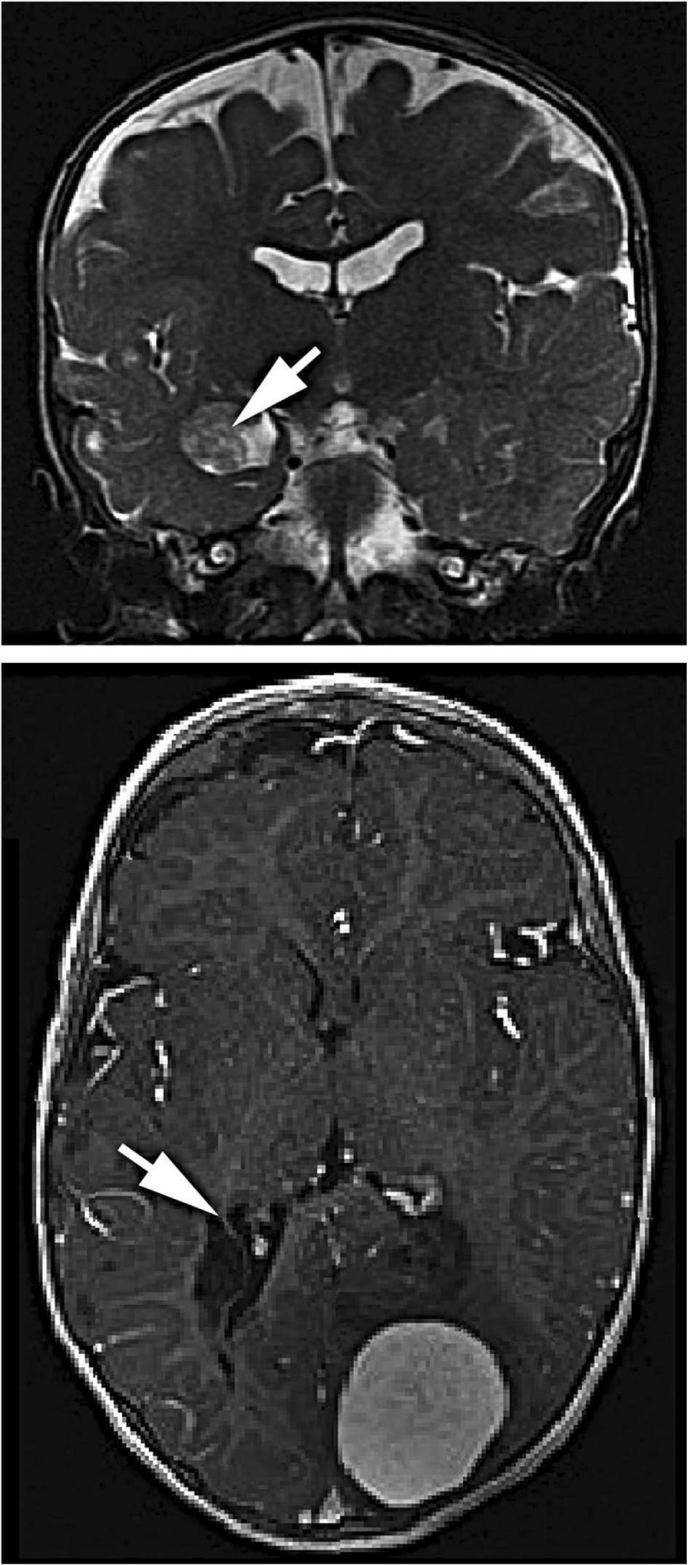
**Magnetic resonance image of the brain.** At 14 months age (upper image, coronal, T2 weighted) an enlarged left temporal lobe and a tumor of the right choroid plexus (arrow) were apparent. At 23 months age (lower image, horizontal, T1-weighted) a 4 cm tumor is present in the left occipital lobe. Periventricular cysts are located adjacent to the right occipital horn (arrow).

This female child was born to a healthy non-consanguineous couple after an uneventful full-term pregnancy. At birth she had numerous slightly raised, hairy melanocytic lesions on the scalp, neck, upper trunk, upper extremities, and hands; the largest was 4–5 cm in greatest dimension (Figure 1). Skin lesions from the neck, scalp, and arm were previously excised and diagnosed as intradermal and compound nevi with congenital features. She had normal height but was macrocephalic (97th percentile head circumference). She had been admitted to hospital numerous times for uncontrolled seizures starting at age 2 months. Magnetic resonance (MR) imaging of the brain and spine was performed at 3.5 months age. T1 weighted images showed a solitary 2 mm focus of increased signal intensity in the right cerebellopontine cistern; this was thought to represent melanin deposition. Cystic lesions, the largest of which was 2.7 cm diameter, with signal characteristics the same as cerebrospinal fluid (CSF) were present around the atria of the lateral ventricles in the right cerebellopontine cistern along with small cysts around the bodies of the lateral ventricles. The left cerebral hemisphere was larger than the right, and parietal white matter volume was abnormally small. The combination of giant congenital nevi along with cerebral melanin deposition led to a clinical diagnosis of NCMS [10]. A CT scan of the head performed at 4 months age during an episode of status epilepticus showed no additional abnormalities. An abdominal sonogram performed the following day showed no abnormalities. Repeat MR imaging showed the presumed melanocytic nodule had increased to 3 mm; it was hyperintense on T1, hypointense on T2, and hyperintense on FLAIR. The periventricular cysts had increased in size and quantity. A new lesion in continuity with the choroid plexus was expanding the temporal horn of the right lateral ventricle. MR imaging of the brain at 21 months age showed a new left occipital brain tumor that was T2 hyperintense and enhanced strongly following administration of gadolinium-DTPA. It grew from 1.6 × 1.5 × 1.2 cm to 4.0 × 3.6 × 3.2 cm 4 months later (Figure 2). A left occipital craniotomy was performed at 24.5 months age. The tumor was not in contact with the meninges. It had a single vascular pedicle. There was an easily identified plane between it and the adjacent brain. The tumor was removed in one piece without complication. MR imaging of the brain 26 months after surgery (51 months age) showed no recurrence of the tumor; a vague region of non-enhancing T2 signal abnormality in the right medial occipital lobe had expanded slightly.

### Histopathological characterization

**Figure 3:**
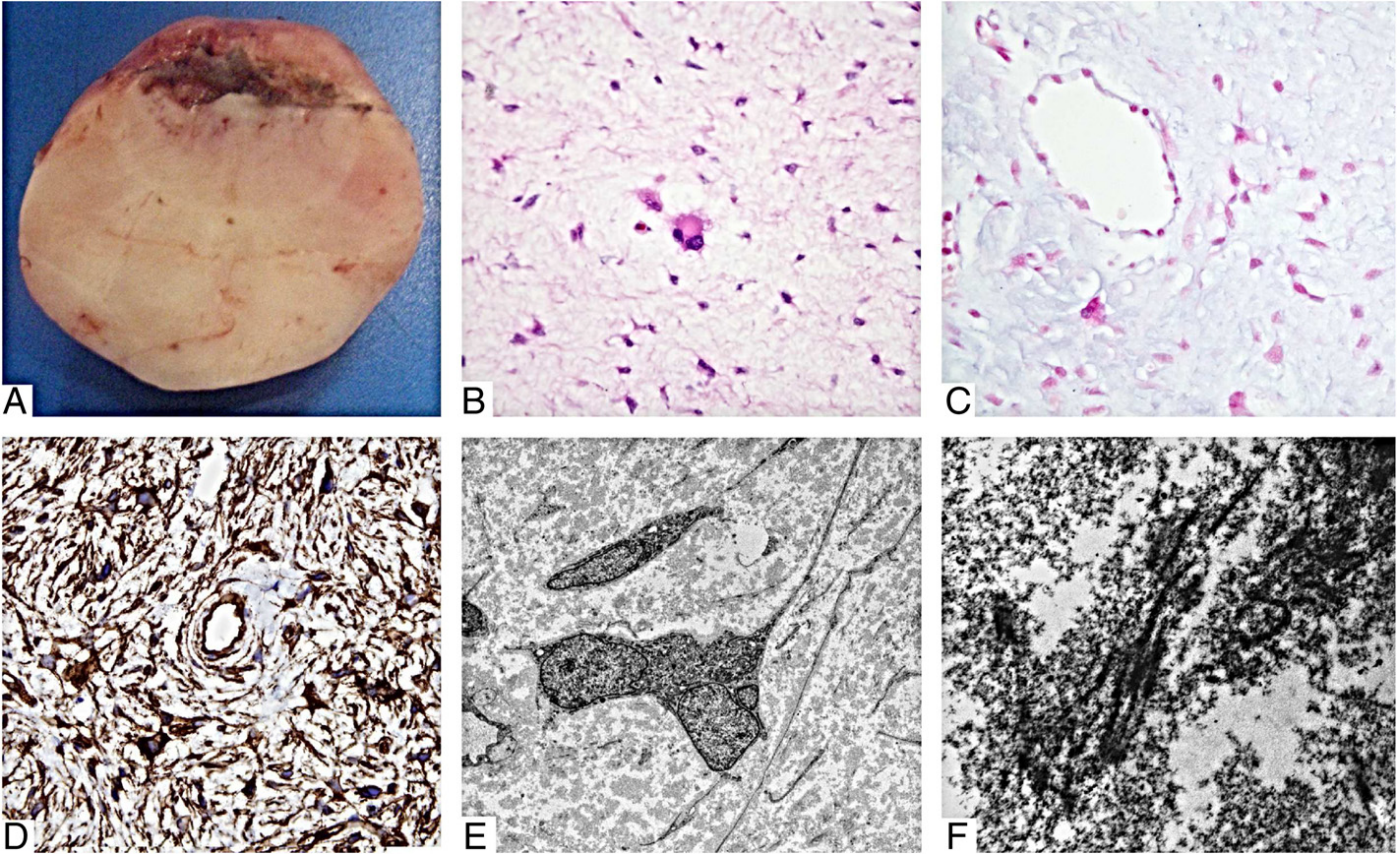
**Macroscopic and microscopic appearance of the tumor. (A)** The firm tumor was easily dissected from the surrounding brain tissue and had a smooth tan-yellow glistening cut surface. **(B)** Microscopic examination revealed a paucicellular tumor consisting of predominantly fusiform cells in a myxoid background. There is moderate nuclear pleomorphism with a multinucleated cell in the center (hematoxylin & eosin). **(C)** The loose myxoid background stained positive for Alcian blue. **(D)** Immunohistochemical staining for CD34 is diffusely positive. **(E)** Electron microscopic examination showed stellate cells with long delicate processes and occasional multinucleation. **(F)** The extracellular material is abundant with clusters of collagen fibers. (Original magnifications: B-C, ×400; D, ×200; E, ×2000; F ×30000).

The tumor was a 4.5 × 3.5 × 3 cm firm tan nodule with a smooth external surface and uniform cut surface (Figure 3A). The brain tumor and skin biopsies were fixed in 10% neutral buffered formalin, dehydrated and embedded in paraffin wax for sectioning (5 uM in thickness). All samples were stained with hematoxylin and eosin for histologic evaluation. Immunohistochemistry was performed using primary antibodies against vimentin (mouse monoclonal, V9, Dako), CD34 (mouse monoclonal, QBEND-10, Dako), alpha B crystallin (mouse monoclonal, G2JF, Novocastra), CD56 (mouse monoclonal, CD564, Dako), D240 (mouse monoclonal, D2-40, Dako), CD99 (mouse monoclonal, 12E71, Dako), Bcl2 (mouse monoclonal, 124, Dako), p53 (mouse monoclonal, D0-7, Dako), Ki67 (mouse monoclonal, MIB-1, Dako), HLA-DR (mouse monoclonal, CR3/43, Dako), Factor 13a (mouse monoclonal, EP3372, Cellmarque), GFAP (rabbit polyclonal, Dako), S100 (rabbit polyclonal, Dako), Collagen IV (mouse monoclonal, C1V22, Dako), pan-cytokeratin (mouse monoclonal, AE1/AE3, Dako), EMA (mouse monoclonal, E29, Dako), CD57 (mouse monoclonal, TB01, Dako), HMB-45 (mouse monoclonal, HMB45, Dako), MART-1/Melan-A (mouse monoclonal, A103, Dako), calretinin (rabbit polyclonal, Cellmarque), neurofilament (mouse monoclonal, 2 F11, Dako), synaptophysin (mouse monoclonal, 27G12, Novocastra), NeuN (mouse monoclonal, A60, Lifespan Biosciences). Antigen retrieval was performed in a Bull’s Eye Decloaking chamber (Biocare Medical, Concord, CA) for 1 minute at 125°C utilizing a Dako pH9 retrieval solution. All antibodies were detected using the Dako Envision system (Dako) and diaminobenzidine precipitation solution. A sample of the tumor was fixed in 2.5% buffered glutaraldehyde, post-fixed in osmium tetroxide, dehydrated in graded ethanol, and embedded in epoxy resin. Semithin sections (0.5 μm) were stained with toluidine blue, and ultrathin sections were contrasted with uranyl acetate and lead citrate then viewed with electron microscopy using a JEM 1010 transmission electron microscope (JEOL Ltd., Tokyo, Japan).

Microscopic examination showed an indistinct interface with the brain parenchyma. The tumor had a diffuse pattern of stellate and elongated cells with delicate processes in a loose myxoid background. There were no pigmented cells. Numerous small, multinucleated cells were evenly distributed within the lesion (Figure 3B). Rarely the cells clustered around blood vessels. Very rare mitotic figures were present and there was no necrosis or endothelial hyperplasia. Focal areas of the extracellular material stained with Alcian blue (Figure 3C). Reticulin staining was negligible. By immunohistochemistry, the cells were positive for vimentin, CD34 (Figure 3D), CD56, D240, Bcl2, CD99 (weak), and P53. A subpopulation of cells was Factor 13a positive. An estimated 5-10% of nuclei were Ki67 positive. Only perivascular cells (likely astrocytes) were positive for glial fibrillary acidic protein (GFAP), S100, and alpha B crystallin. Blood vessel walls were positive for E cadherin and collagen IV. There was no immunoreactivity for cytokeratin (AE1/3), epithelial membrane antigen (EMA), CD57, HMB-45, MART-1/Melan-A, calretinin, neurofilament, synaptophysin, or NeuN. Scattered HLA-DR positive cells were likely infiltrating microglia. Electron microscopic examination showed fusiform cells with abundant rough endoplasmic reticulum and prominent intermediate filaments but no specific secretory organelles or obvious intercellular junctions. The cells lacked a well-defined basement membrane and were surrounded by a flocculent extracellular material with rare clusters of striated collagen bundles (Figure 3E, 3F). The diagnostic categorization arrived at locally and supported by external consultation was myxoid mesenchymal brain tumor of uncertain growth potential.

### DNA extraction and sequencing

Genomic DNA was extracted from formalin-fixed paraffin-embedded samples of the intracranial tumor, an excised cutaneous nevus lesion, and grossly unaffected skin. DNA was also extracted from swabbed buccal cells of the patient and both of her parents. DNA was quantitated using the Qubit 2.0 fluorometer (Life Technology). Focused deep sequencing of 10 ng of genomic DNA extracted from the above samples was performed using the Ion AmpliSeq Cancer panel (Life Technology). This includes PCR primers covering 739 potential cancer-related hotspot mutations in 46 genes including *KRAS, NRAS, BRAF, PIK3CA, and IDH1* [11]. This technology permits the interrogation of genetic alterations including mutations and insertions/deletions, even minor alleles in complex samples, in suboptimal specimens including formalin-fixed paraffin-embedded tissues. Processing of all samples was performed according to the manufacturer’s protocol. Construction and enrichment of the emulsion PCR library was performed using the Ion OneTouch instrument. Sequencing was done on Ion 314 and Ion 316 sequencing chips using the Ion Torrent Personal Genome Machine (Life Technology) following the manufacturer’s protocol. Data analysis including alignment to hg19 human reference genome, base calling, and identification of variants was done using the Ion Variant Caller (version 2.2). Somatic variants from the brain tumor and the melanocytic nevus were identified after filtering out germline changes identified from the patient’s buccal swab. Allele frequency of a variant is calculated by dividing the number of variant reads by total reads in the same nucleotide position. All variants are covered by a minimum of 500 reads.

The panel identified a missense mutation (chr1:115258745; c.37G > C) in *NRAS* that results in a p. G13R amino acid substitution. The allelic frequency of the nucleotide change in the brain tumor and the cutaneous nevus (58% and 33% respectively) is consistent with a heterozygous mutation (Table 1). The same change was observed at an allelic frequency of 4.8% in the normal skin and 2.7% in the buccal swab of the patient. Deep sequencing of DNA extracted from the buccal swabs from both parents did not reveal *NRAS* mutations. A *BRAF* somatic nucleotide change (chr7: 140481441; c.1397G > T) resulting in amino acid substitution p. G466V was detected only in the nevus at an allelic frequency of 32%. A single nucleotide polymorphism (SNP) resulting in a missense mutation (chr7: 116411990; c. 3209C > T; p.T1010I;) was found in the *MET* gene of the child’s tumor and buccal swab as well as in the buccal swab of the father.

**Table 1:** **Allelic frequency of nucleotide change in** ***NRAS*** **and** ***BRAF*** **from deep amplicon sequencing of pathology specimens and buccal swabs**

**Gene**	**NRAS**	**BRAF**
Chromosome position (hg19)	1:115258745	7:140481411
Nucleotide change	c.37G > C	c.1397G > T
Amino acid change	p. Gly13Arg	p. Gly466Val
	Allelic frequencies (%)	
Mesenchymal brain tumor	58.0	0.0
Melanocytic nevus	33.0	32.0
Normal skin	4.8	0.0
Buccal swab	2.7	0.0
Mother buccal swab	0.0	0.0
Father buccal swab	0.0	0.0

## Discussion and conclusions

Neurocutaneous melanosis (NCMS), first described by Rokitansky in 1861 [12], is a rare congenital disorder consisting of multiple large melanocytic cutaneous nevi and melanocytic proliferations in the leptomeninges [13]. Malformative lesions of the posterior fossa have rarely been described [14,15]. Recent genetic findings have clarified the pathogenesis of NCMS and large/giant congenital melanocytic nevi [7,16]. Single postzygotic mutations of *NRAS* codon 61 and associated mosaicism are responsible for the majority of NCMS cases [5] and large/giant congenital melanocytic nevi [17]. The same mutation is common in congenital melanocytic nevi [18]. The *NRAS* p.G13R somatic mosaicism in this patient is unusual and, to our knowledge, is the first instance reported in association with NCMS. The presence mosaicism in combination with the absence of mutation in the parents suggests this mutation likely occurred after conception. This is consistent with the mosaicism hypotheses for NCMS and other phakomatoses [19]. This child does not show any dysmorphic features associated with germline mutations in *NRAS*, which are usually similar to Noonan syndrome [20]. *NRAS* mutations are considered important in the genesis of melanoma. NRAS activates four major signaling pathways including RAF-MEK-ERK, RalGDS, PI3K-AKT/PDK1, and PLC/PKC [21]. Mutations affecting codons 12, 13 and 61, lead to constitutive activation of RAS GTPase in the absence of growth factor signaling and ultimately neoplastic growth. The specific NRAS G13R mutation identified in this case has been rarely found in melanomas of the skin [22-24] and esophagus [25] as well as in 1/27 patients with large congenital melanocytic nevi [26,27]. In melanoma the most common *NRAS* (Q61R, Q61L, Q61K) and *BRAF* (V600E, V600K) mutation sites and substitutions differ from those found in this patient [28].

In addition to NCMS, this child had a low-grade mesenchymal brain tumor, which itself is very rare. Typically children with NCMS do not develop sarcomas, although one rhabdomyosarcoma was described in a congenital giant nevus [29]. Children with NCMS are reported to develop choroid plexus papilloma and meningioma [5]. Primary sarcomas of the brain represented only 0.36% of brain tumors in one very large series [30] and 0.7% of all sarcomas in another series [31]. Most likely arise from the meninges or blood vessels; among them are rhabdomyosarcoma, fibrosarcoma, leiomyosarcoma, and angiosarcoma. Primary non-meningeal myxoid mesenchymal intracranial tumors are especially rare. Reported cases include two low-grade fibromyxoid sarcomas [32,33] and one myxofibrosarcoma, the diagnosis of which was based on fluorescent in situ hybridization (FISH) analysis of the *FUS/CREB3L2* translocation [34]. Both of these tumors have some features similar to the reported child’s brain tumor. It is important to emphasize that the tumor had no morphologic or immunophenotypic features of melanoma. The relatively low-grade appearance of the brain tumor presented herein is discrepant with its rapid growth. The rapid increase in size could be explained by expansion of the myxoid extracellular material rather than neoplastic cell proliferation.

The heterozygous state of *NRAS* in the melanocytic nevus and the brain tumor combined with additional novel somatic mutations suggest cooperative involvement of oncogenic pathways in the brain tumor and especially in the skin lesions (*BRAF* p.G466V). Only a single sarcoma (a rhabdomyosarcoma) has been described with the *NRAS* p.G13R mutation [35]. Somatic mosaics of *NRAS* mutations in almost identical protein regions (G12D, G12S, G13D) have been described in relation to juvenile myelomonocytic leukemia [36,37]. Note that neurofibromin is a major regulator of the NRAS pathway [38]. *NF1* is not assessed in the AmpliSeq Cancer Panel and we did not attempt direct sequencing. However, given that the documented abnormalities are distal to neurofibromin signaling, a mutation in NF1 is not necessary to explain this child’s phenotype.

A germline SNP was found in the *MET* gene of this child (inherited from her father who is not known to have any neoplastic disease). The *MET* gene encodes the receptor for hepatic growth factor/scatter factor (HGF/SF) and appears to be involved in cell motility, proliferation, and invasiveness [39]. Mice that overexpress HGF/SF overstimulate the MET pathway and develop melanosis in the central nervous system and patterned hyperpigmentation of the skin similar to NCMS [40]. These mice also develop fibrosarcoma and rhabdomyosarcoma [41]. MET has been detected by immunohistochemistry in optic canal nevus cells from a child with NCMS [42]. The p.T1010I variant has been identified in thyroid carcinomas [33], neuroendocrine carcinoma (NEC) of lung [43], a pleomorphic xanthoastrocytoma case [11], and has been implicated as a risk factor for familial colorectal cancer [44]. One early report described this change in MET as capable of altering signaling in NEC [45], however more recently this variation was not shown to alter c-Met phosphorylation in NEC [46], nor does it seem capable of transforming the Ba/F3 pro-B lymphocyte cell line [47]. To summarize, the significance of the *MET* SNP in this child is unclear.

Hemimegalencephaly has been associated with other neurocutaneous syndromes including epidermal nevus syndrome, proteus syndrome, hypomelanosis of Ito, and neurofibromatosis-1 [48,49]. Recently, de novo somatic mutations with mosaicism in the PI3K-AKT3-mTOR pathway were shown to cause hemimegalencephaly [50]. NRAS is known to have direct interaction with PI3K [21]. Given that this child had no mutation in *PI3K*, the *NRAS* mosaicism is likely the explanation for the hemimegalencephaly.

## Conclusion

In summary, development of a primary intracerebral mesenchymal neoplasm in a child with NCMS and hemimegalencephaly can likely be explained by specific *NRAS* mutant mosaicism possibly in combination with a *MET* germline variation, which together constitute a unique combination. This case highlights the importance of DNA analysis from multiple sites, as well as from parents, in individuals with complex disease states. The presence of an *NRAS* somatic mosaic supports the hypothesized developmental pathogenesis of NCMS. Further exploration of the role of *NRAS* and *MET* in development of the neural crest derived pigment cells will be of interest.

## Consent

Parents provided explicit consent for genetic tests and use of photographs.

## Abbreviations

BRAF: B-Raf proto-oncogene, serine/threonine kinase
DNA: Deoxyribonucleic acid
FISH: Fluorescent in situ hybridization
MET: MET proto-oncogene, receptor tyrosine kinase
MR: Magnetic resonance
NCMS: Neurocutaneous melanosis
NRAS: Neuroblastoma RAS viral (v-ras) oncogene homolog
PCR: Polymerase chain reaction
